# Ethics choices during the Human Genome Project reflected their policy world, not ours

**DOI:** 10.1016/j.xgen.2025.100841

**Published:** 2025-05-14

**Authors:** Jonathan E. LoTempio, Christopher R. Donohue, Jonathan D. Moreno, Robert Cook-Deegan

**Affiliations:** 1 University of Pennsylvania Perelman School of Medicine, Philadelphia, PA, USA; 2 National Human Genome Research Institute, Bethesda, MD, USA; 3 Arizona State University School for the Future of Innovation in Society, Tempe, AZ, USA

## Abstract

Since human genomic data produced in the 1990s are still a significant part of the reference genome, decades-old decisions pertinent to the creation of these data persist. Here, we discuss how historical documents illustrate the 1990s policy and legal environment and how they affected ethical choices in the Human Genome Project (HGP). These documents inform current controversies about informed consent and how IRBs review similar protocols today. Finally, we discuss how this informs active work in large reference pangenome efforts.

## Main text

### Introduction

The consent form used during the Human Genome Project (HGP) is at the center of an ethics controversy.^1^^,^^2^ Questions influencing this consent form address respect for people, the sometimes-opposing roles of public and private science, and the human experience of participating in science as researcher and participant. To understand this interplay, we find two historical documents particularly instructive. They include a government guidance document from July 1996 and an ethics panel decision from February 1998. Here, we discuss these documents and aim to answer key questions pertinent to this ethics controversy.

### Historical document 1: The DOE-NIH guidance on human participant issues

In 1996, the International HGP was ready to transition from gene mapping, that is, locating genetic elements on chromosomes, to the large-scale sequencing of genomes—determining the order of bases in DNA constituting the genome. At the end of the chromosome mapping phase, before sequencing began, samples used for mapping were deemed to be inappropriate for the reference sequence, in part because they were derived from a known researcher.^1^^,^^3^

Accordingly, investigators prepared to recruit new participants to donate samples anonymously. Since this was the first instance of samples being explicitly collected for a high-quality genome destined for the public domain, Dr. Ari Patrinos, the associate director of science for the Biological and Environmental Research program of the US Department of Energy (DOE), and Dr. Francis Collins, the director of the US National Institutes of Health (NIH) National Center for Human Genome Research (NCHGR), jointly (DOE-NIH) issued guidance on recruiting volunteers to donate samples for the reference genome sequence. They outlined six points for consideration, including four general risks.

To enhance human participant protections, they recommended the following:

(1) The public nature of the project could lead to personal distress, insurance or employment discrimination, or unwanted notoriety for the human participants. There are also risks that could not be known at the outset of the projects. The risk of these harms is low but can be reduced further through confidentiality and ensuring that “the number of donors is [not] limited to a very few individuals.…”
(2) The genome is fundamentally identifiable but not readily identifiable because “the technology that would allow the unambiguous identification of an individual from his/her DNA sequence is not yet mature.” This promotes two-way confidentiality where the investigators do not know the donors and the donors do not know whether their sample was used, which protects them against a loss of anonymity through technological advancement.
(3) While the backgrounds of any donor will not matter, there is no scientific reason to not select donors from a diverse pool of people. Women have been historically underrepresented, and even though half of a donor’s DNA is from their mother, “perceptions [of exclusion] are not to be dismissed.” Furthermore, including research-study staff may increase the chance of loss of confidentiality and be perceived as elitist.
(4) The informed consent process must address the following points:
  a. The meaning of confidentiality and privacy of information in the context of large-scale DNA sequencing and how these issues will be addressed.
  b. The donor cannot later withdraw the libraries made from his/her DNA or his/her DNA sequence information from public use because any links between the sample and donor will be broken to preserve anonymity.
  c. No information of clinical relevance will be provided to the donor or her/his family.
  d. The possibility of unforeseen risks.
  e. The possible extension of risk to family members of the donor or to any group or community of interest (e.g., gender, race, and ethnicity) to which a donor might belong.

A fifth point stipulated that the protocol for recruiting volunteers be reviewed and approved by an institutional review board (IRB), which should consider the points above that are unique to creating a human genome for the public domain. Finally, a sixth point explained that not all existing samples were appropriate to use in creating the reference sequence and any continued use would be decided through case-by-case review.

### Historical document 2: The Roswell Park Cancer Institute IRB decision

One of the groups funded to collect samples for the HGP was based at Roswell Park Cancer Institute (RPCI) in Buffalo, NY, and led by Dr. Pieter de Jong. His research group processed the samples for sequencing into DNA segments cloned in bacterial artificial chromosome (BAC) libraries that could be replicated and sent around the world. A second group at RPCI was responsible for participant recruitment.

The RPCI groups submitted a protocol to the IRB, which was approved in February 1997. The opportunity to participate was advertised along with a cover story in the *Buffalo News* on March 23, 1997.^4^ The story by journalist Henry L. Davis began “Wanted: Two Buffalo area residents – a man and a woman – willing to donate their DNA for the world’s biggest science project – deciphering the genetic blueprint for human life. The pay is small; the benefits – to humanity – are great.”

As specified in the IRB protocol, the recruitment team conducted an hour-long informed consent conversation, collected signed forms, designated unique identifiers, and paid cash compensation (with no paper trail) for donors’ time. To preserve two-way confidentiality, the informed consent documents were kept (and locked up) by a coordinator separate from the team making the BAC clones.

In November 1998, the IRB was asked to revisit the informed consent form because only one volunteer’s BAC clones were of high quality and thus more suitable for creation of a reference sequence. Dr. de Jong asked the IRB to review “two sentences” of the consent form because the amount of human sequence generated from that one donor had expanded well beyond the initial expectation. This donor was given the unique identifier RP11 (sometimes RPC11, sometimes RPCI-11). Archival materials show that Dr. de Jong informed the IRB that RP11 might end up constituting 60%–90% of the public reference sequence, while the consent form said the following:

This means that even when the whole human DNA sequence is known, it will have been derived from DNA from a large number of people. If we use the blood you donate to generate a BAC library, then we expect that no more than 10% of the eventual DNA sequence will have been obtained from your DNA.

The question posed to the IRB was “whether or not there should be an attempt to contact all ten (10) of the male donors and have them sign an amended consent form in which the wording of the second sentence changes from ‘then we expect that no more than 10%.…’ to ‘then up to 60[%]–90%.’”

**Figure 1:**
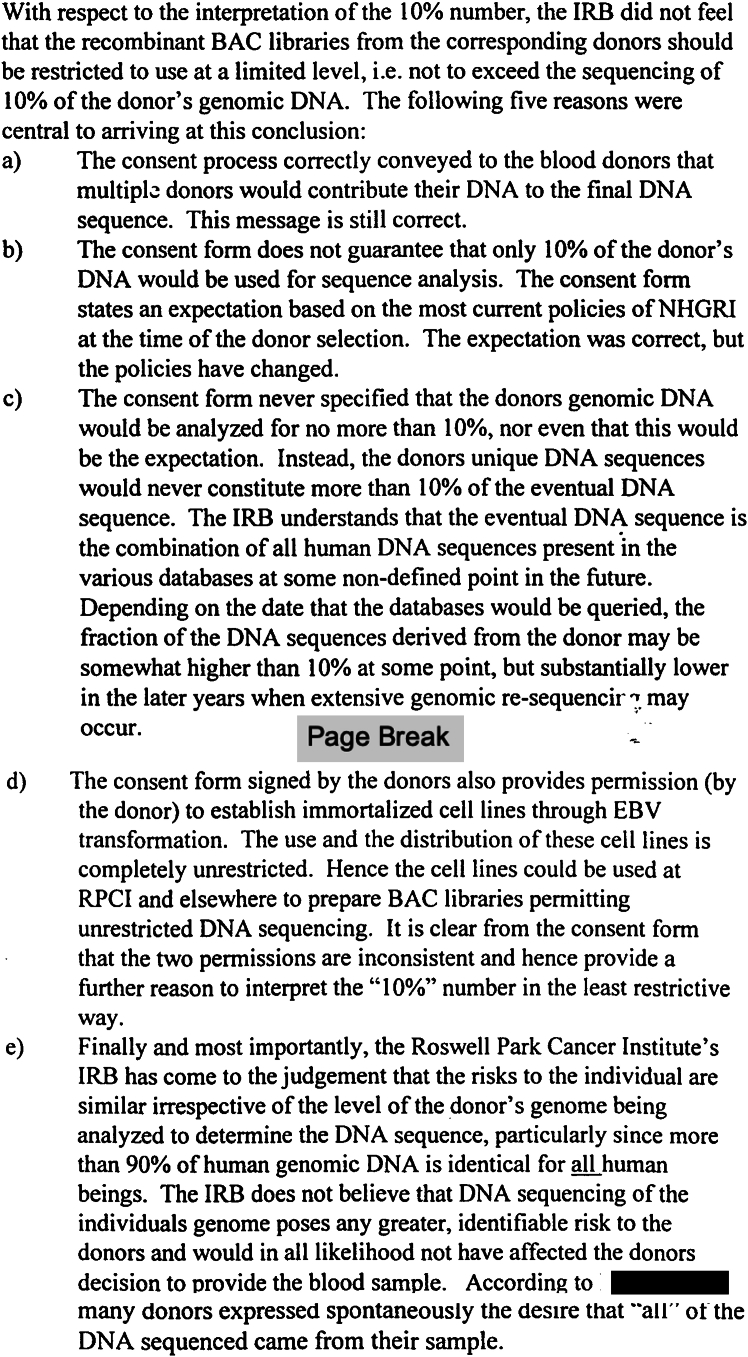
Excerpt from Roswell Park IRB decision Reproduction of the 1998 Roswell Park Cancer Institute IRB justification for continued use of RP11 without recontact, titled “Administrative Matters — excerpt of draft from the 10/27/98 IRB Meeting.” One name is redacted for privacy. Break between pages 2 and 3 of the original archival document is emphasized.

The IRB was unanimous in concluding that the donors should not be recontacted. They provided five reasons, summarized here (see the complete form in Figure 1).

(1) The consent process explained that multiple donors would be used, and indeed they were.
(2) The consent process did not guarantee, but rather “expected,” that no more than 10% of the sequence would be from any one individual.
(3) The percentage of any one donor would change over time as participants were added.
(4) The consent for cell lines was “consent for anything,” including whole-genome sequencing, and was thus inconsistent with limits on the amount of sequence production from any one person’s DNA.
(5) The risks of determining that a particular participant was used to constitute the reference sequence did not increase much between 10% and a higher percentage.

### Analysis

#### Convergence and divergence between the joint guidance and the IRB decision

The scientific arguments outlined by the IRB rested on the practicalities of generating a reference sequence composed of different numbers of people at different points, with the number of participants likely increasing over time. The risk of identifying RP-11 as outlined in the DOE-NIH joint guidance did not increase greatly between 10% and 60%–90% because 10% was still a substantial fraction, much more than enough to indicate that person’s DNA was in the reference (https://web.archive.org/web/19980204150937/http:/www.nhgri.nih.gov/Grant_info/Funding/Statements/RFA/human_subjects.html).

The consent form, as a whole, was consistent with the outcome of one person constituting most of the reference sequence. The spirit of the consent can be variously interpreted.^1^^,^^2^ However, consent to have a cell line produced involves a conversation that would now be called broad consent.^5^^,^^6^ The IRB concluded that the breadth of permission for creation of cell lines implied permission for sequencing those cell lines entirely, so the informed consent permitted whole-genome sequencing.

The high fraction of sequence from one person in the reference sequence would thus be covered by the informed consent to create cell lines, and the 10% “expected” fraction of the composite genome was not decisive in the informed consent. Notes from the IRB meeting indicate that the informed consent generally took one hour, and most participants were simply excited to contribute to the famous HGP. Indeed, some donors expressed a wish that their samples be used for the entire reference sequence.

Honoring the promise of anonymity, and protecting the elaborate process for ensuring it, was deemed more important than informing a participant of higher, but still unknown, total level of contribution with only marginal increased risks. The two-way confidentiality was clearly the primary concern of the IRB, and recontacting participants would break this.

The IRB was silent on another point raised in the joint guidance: that incentives for re-identification would increase if one or a few people’s DNA made up most of the reference. And indeed, the reason for the current controversy centers on that one person’s contribution, the person who donated the RP-11 sample. One feature of this controversy is whether that person deserves recognition and credit.^1^

#### IRBs: Meetings and decisions

It is important to clarify the scope and function of an IRB. In the US, an IRB is an independent unit at a research institution composed of people at that institution in accordance with federal regulations.^7^ These regulations, the US Federal Policy for the Protection of Human Subjects, are known as the Common Rule because they are “common” across 20 signatory departments and agencies.^8^ The first federally regulated IRBs were established following the passage of the National Research Act in 1974.^9^

IRBs make decisions on the “ethical acceptability of proposals for human research.”^7^ They do not make decisions on the quality of projects. That sort of assessment only happens at the time of grant application review or publication peer review. IRB decisions are final, but as new questions arise, they may be asked to address them. IRB chairs and human-participant-research ethics specialists often meet with investigators.

**Figure 2:**
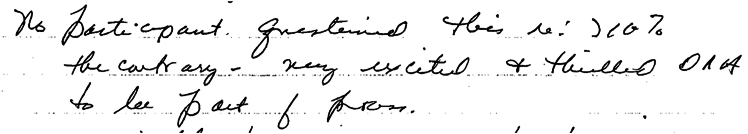
Excerpted notes from Roswell Park IRB meeting “No participant questioned this re 10%?” “The contrary - very excited and thrilled DNA to be part of process.”

One such meeting was held on October 22, 1998, to plan for the November IRB meeting, which Pieter de Jong requested to review again the recruitment protocol in light of the reference sequence relying heavily on just one volunteer’s sample. Roswell Park investigators, including the BAC library team and the recruitment team, along with NIH program staff, met with the director of the Roswell Park IRB and the director of “Institutional Protocol.” At that meeting, they discussed the consent form, the consent process, and procedural realities. All of the 5 points, with the exception of point 3 (percentage of DNA from the RP11 sample), were discussed at this meeting. The practicalities or possibilities of recontacting were discussed, as were the increased risks to the participants if recontact were attempted. As per handwritten notes from the time, participants did not raise concern about the 10% fraction and were excited to have their DNA be part of the genome (Figure 2).

Finally, at this meeting the question “why a fast track?” was posed regarding the decision to not delay the HGP sequencing effort in order to reduce incentives to re-identify RP-11. The answer was, essentially, to discover more genes and help more people. The final sentence of the IRB decision stated plainly that “unwarranted delay would be unethical. Termination of further sequencing of [RP11] would result in such a delay.”

Ultimately, the IRB decided that given the extraordinary measures originally taken to anonymize these donors and given that there were no provisions made for ever recontacting the donors, the likelihood of recontacting all 10 of them was low. More importantly, any attempts to locate these individuals, either through the telephone directory or newspapers, raised the risk of both arousing public suspicion and of contacting the wrong people, including donors’ relatives, thereby compromising the anonymity of the donors. Such attempts would place them at greater risk of being identified as the possible donor of blood from which the (RP11) library was derived. The donors were specifically told that every effort would be made to prevent this.

#### Consent, anonymity, confidentiality, and identity

Were participants misled in the consent process? Based on our experiences staffing and chairing IRBs, and our academic work, we think that they were not.^6^^,^^10^^,^^11^ Why?

The historical documents reveal thoughtful, albeit imperfect, consideration of research ethics. The investigators, approved by the IRB, were empowered to continue work and prevent the participant from being identified, rather than break confidentiality and recontact the male volunteers to clarify consent. Why were anonymity and confidentiality top of mind in the late 1990s?

Loss of anonymity and confidentiality was considered to be the major risk to participants in this phase of the HGP. There were few protections for people and their genetic information. In the intervening years, the US legal system has created new protections through both the Genetic Information Nondiscrimination Act (GINA) in 2008 and the Affordable Care and Patient Protection Act (ACA, Obamacare) in 2010.^12^ The 21st Century Cures Act of 2016 mandates that certificates of confidentiality cover participants in NIH-funded research. These laws mitigate risks of re-identification through legal and policy protections (https://grants.nih.gov/policy/humansubjects/coc.htm). In effect, American society has legislated away some of the concerns that shadowed the dawn of genomics.

The consent documents used during the HGP reflected the importance of anonymity, given the paucity of protections that participants had at the time. Current protections build, in part, on that historical experience. One need only look to later consent forms available from the National Human Genome Research Institute to see how the emphasis on double-blind confidentiality is replaced by exhortations to keep one’s participation to one’s self (https://www.genome.gov/about-genomics/policy-issues/Informed-Consent/Sample-Consent-Forms). The Informed Consent for the 1000 Genomes Project, for example, reads as follows: “Any of these things [risks] would require that the person trying to link the information to you knew that you participated in the project. For this reason, to minimize these risks, you may wish to limit the number of people you tell about your participation.”

In a pre-GINA, pre-ACA world, anonymity and confidentiality were paramount. The creation of these laws and related policies reduced some risks from the HGP era. In the intervening years, the world has learned that Craig Venter, Peter DeJong, Leonid Peshkin, Steve Pinker, George Church, Bishop Desmond Tutu, and other high-profile personalities have participated in genomics in public ways (https://nebula.org/genomic-nft/).^1^^,^^13^^,^^14^ Others, including James Watson, J. Craig Venter, Bishop Desmond Tutu, Misha Angrist, and other participants in the Personal Genome Project (including one of us), have put their entire genomes on the internet (https://grants.nih.gov/policy/humansubjects/coc.htm). Whether the caution suggested by the 1000 Genomes Project is as necessary now as it was then is an outstanding question. Identification, moreover, has the added benefit of the possibility of clarifying consent, and intent, at future times.

Accordingly, the level of protection from re-identification is an active debate. The 21st Century Cures Act conferred certificates of confidentiality and reduced risks of state-coerced re-identification. GINA and the ACA proscribed genetic discrimination for employment and health insurance, while other risks persist. However, some projects, such as the Personal Genome Project, enable individuals to retain access to their entire genome sequence and to share publicly. Thus, norms may be shifting in at least some circumstances. Legal protections have reduced risks but not eliminated them. The Human Pangenome Reference Consortium (HPRC), for example, will share only gender and site of collection for samples and sequences made public, reflecting this persistent concern about risks of re-identification (forthcoming work from many including some of the authors here).^15^

It is important to note that several points addressed in the 1996 joint guidance have been ignored. Considerations of identity and perceptions of exclusion, specifically around the inclusion of women or the concern of including scientists over non-scientists, have not received much attention until recently. The HPRC aims to reflect more of the human family than is possible in the list of predominantly male and notable individuals mentioned. The HPRC is also supporting its ethical, legal, and social implications (ELSI) team to study when it is appropriate to include people whose identities are known to have their entire genomes included in public pangenome resources.

### Conclusions

The person designated RP11 participated in an important project at a time when protections for genomic data were limited. To accommodate the legal and policy environment of that time, two-way confidentiality was deemed paramount. Preservation of this confidentiality, and thus protection from harms (non-maleficence and beneficence), outweighed clarification of informed consent (respect for autonomy).

The volunteer whose DNA became sample RP11 participated in a project that had detailed documents to inform him of what it meant to be part of a freely available human reference sequence. Volunteer RP11 did not participate in a project that objectified an unsuspecting individual. In short, the investigators were respectful, thorough, and consulted with the IRB when appropriate. In 2025, we have stronger protections that reduce the need for two-way confidentiality while promoting non-maleficence and respect for participant autonomy in research. This better-protected research environment is partly a result of subsequent efforts to legislate protections, a policy response to a time and place with weaker protections.

It would be ethically straightforward if someone who thought that they were the participant known as RP11 wanted to identify himself. As with any project in our better protected world, he can simply choose to do so. However, that choice to come forward belongs to the participant.

## Acknowledgments

The authors are grateful to the National Human Genome Research Institute for preserving such robust archival materials, without which this research would not have been possible. J.D.M. has been supported by the David and Lyn Silfen Family University Professorship and is the Silfen Family University Professor Emeritus.

## Author contributions

J.E.L., Jr., C.R.D., J.D.M., and R.C.-D. were involved in the conceptualization of this work. J.E.L., Jr. and C.R.D. conducted the archival investigation. J.E.L., Jr. and R.C.-D. wrote the manuscript. C.R.D. and J.D.M. reviewed and edited the manuscript.

## Declaration of interests

The authors declare no competing interests.
